# Carbapenem-Resistant *Klebsiella pneumoniae* Clinical Isolates: In Vivo Virulence Assessment in *Galleria mellonella* and Potential Therapeutics by Polycationic Oligoethyleneimine

**DOI:** 10.3390/antibiotics10010056

**Published:** 2021-01-08

**Authors:** Dalila Mil-Homens, Maria Martins, José Barbosa, Gabriel Serafim, Maria J. Sarmento, Rita F. Pires, Vitória Rodrigues, Vasco D.B. Bonifácio, Sandra N. Pinto

**Affiliations:** 1 iBB—Institute for Bioengineering and Biosciences, Department of Bioengineering, Instituto Superior Técnico, Universidade de Lisboa, Av. Rovisco Pais, 1049-001 Lisboa, Portugal; dalilamil-homens@tecnico.ulisboa.pt (D.M.-H.); mald.martins@campus.fct.unl.pt (M.M.); josembarbosa@tecnico.ulisboa.pt (J.B.); gabriel.serafim@tecnico.ulisboa.pt (G.S.); ritafpires@tecnico.ulisboa.pt (R.F.P.); 2 J. Heyrovský Institute of Physical Chemistry of the Czech Academy of Sciences, Dolejskova 3, 18223 Prague, Czech Republic; maria.sarmento@jh-inst.cas.cz; 3 Secção de Microbiologia, Laboratório SYNLAB-Lisboa, Grupo SYNLAB Portugal, Av. Columbano Bordalo Pinheiro, 75 A, 2° Andar, 1070-061 Lisboa, Portugal; vitoria.rodrigues@synlab.pt

**Keywords:** *Klebsiella pneumoniae*, KPC and OXA-48-like carbapenemases, *Galleria mellonella* infection model, linear oligoethyleneimine hydrochloride

## Abstract

*Klebsiella pneumoniae*, one of the most common pathogens found in hospital-acquired infections, is often resistant to multiple antibiotics. In fact, multidrug-resistant (MDR) *K. pneumoniae* producing KPC or OXA-48-like carbapenemases are recognized as a serious global health threat. In this sense, we evaluated the virulence of *K. pneumoniae* KPC(+) or OXA-48(+) aiming at potential antimicrobial therapeutics. *K. pneumoniae* carbapenemase (KPC) and the expanded-spectrum oxacillinase OXA-48 isolates were obtained from patients treated in medical care units in Lisbon, Portugal. The virulence potential of the *K. pneumonia* clinical isolates was tested using the *Galleria mellonella* model. For that, *G. mellonella* larvae were inoculated using patients KPC(+) and OXA-48(+) isolates. Using this in vivo model, the KPC(+) *K. pneumoniae* isolates showed to be, on average, more virulent than OXA-48(+). Virulence was found attenuated when a low bacterial inoculum (one magnitude lower) was tested. In addition, we also report the use of a synthetic polycationic oligomer (L-OEI-h) as a potential antimicrobial agent to fight infectious diseases caused by MDR bacteria. L-OEI-h has a broad-spectrum antibacterial activity and exerts a significantly bactericidal activity within the first 5-30 min treatment, causing lysis of the cytoplasmic membrane. Importantly, the polycationic oligomer showed low toxicity against in vitro models and no visible cytotoxicity (measured by survival and health index) was noted on the in vivo model *(G. mellonella)*, thus L-OEI-h is foreseen as a promising polymer therapeutic for the treatment of MDR *K. pneumoniae* infections.

## 1. Introduction

The widespread use of antibiotics in clinics caused an increased frequency of multidrug-resistance bacteria mainly due to bacterial mutations [1]. In particular, the emergence of resistance to last resource antibiotic treatment options (including carbapenems) has contributed to the limitation of effective therapeutics. Recent reports revealed a weak pipeline for novel antibiotics. From all the compounds under development, very few target infections were caused by Gram-negative bacteria [2,3,4]. This is clinically relevant, as Gram-negative bacteria infections are significantly more lethal compared to those caused by the Gram-positive [3]. Several factors contribute to the scarcity of new antibiotics, market failure being the most relevant. As result of a low return investment, pharmaceutical companies lack incentives for novel antibiotics development. Antibiotics are fast-acting drugs (limiting patient requirements to a small-time window) and the use of novel antibiotics is often reserved since, ultimately, an unpredictable resistance may occur [1,5]. Therefore, the development of novel and efficient antimicrobial agents is of utmost priority. 

*Klebsiella pneumonia*, a pathogen of the *Enterobacteriaceae* family, is resistant to last resource antibiotics and is the source of some of the most complicated hospital-acquired infections [6,7,8]. Resistance to carbapenem in *K. pneumonia* poses a significant threat to patients in hospitals as this organism can cause life-threatening infections such as pneumonia, bloodstream infections and sepsis [9]. Several factors are associated with the acquisition of *K. pneumonia* KPC(+) and OXA-48(+) bacteria, including prolonged hospitalization, infections caused by medical devices (including contamination of ventilators and catheters) and overuse of antibiotics (e.g., carbapenems). Carbapenems-resistant *K. pneumonia* bacteria are capable of inactivating carbapenems via the production of carbapenemase enzymes. Several carbapenemases have been identified and categorized into classes. The ambler classes A (KPC, plasmid-mediated clavulanic acid-inhibited β-lactamases) and D (OXA-48, expanded-spectrum oxacillinase) categories are considered relevant carbapenemases, being highly resistant to all β-lactam molecules, including carbapenems [10]. 

To investigate the in vivo relevance of MDR *K. pneumonia* infection, we obtained different KPC(+) and OXA-48(+) isolates and determined their virulence using *Galleria mellonella,* a caterpillar model of infection. The success of MDR *K. pneumonia* infections depends, “among other factors”, on the ability of the pathogen to escape the host’s defense mechanisms. The larvae of the greater wax moth *G. mellonella* have been successfully employed as a model host to study virulence of human pathogenic agents, including several human pathogens, and to investigate the efficacy of therapeutic drugs [11,12,13,14]. *G. mellonella* possess only an innate immune system (that includes melanization, hemolymph, and several antimicrobial peptides). However, this is enough to offer powerful resistance to microbial infections [15]. Additionally, their innate immune system shares a high degree of structural and functional homology with the innate immune systems of mammal’s [16]. Thus, evaluation of *G. mellonella* responses to *K. pneumonia* isolates infection can provide indication of the mammalian response to these pathogens.

Very few treatment options are available for patients infected with *K. pneumoniae* producing KPC or OXA-48-like carbapenemases and are often limited to administration of multiple antibiotic therapies and to colistin [17,18]. In the light of this, in this study, we report the use of a polycationic synthetic oligomer, linear oligoethyleneimine hydrochloride (L-OEI-h), as an antimicrobial agent for the treatment of *K. pneumonia* KPC(+) and OXA-48(+) bacterial infections. We have previously reported the synthesis and biocidal activity of L-OEI-h against *Streptococcus aureus* and *Escherichia coli* [19]. Herein, we evaluate L-OEI-h antibacterial activity against MDR bacteria clinical isolates, namely *K. pneumoniae*, and investigate the underlying mechanism of action, which, as found for other polycationic antimicrobial agents, might involve disruption of the cell wall and/or the disintegration of the cytoplasmic membrane [10]. 

## 2. Results

### 2.1. Evaluation of K. pneumoniae Virulance in G. mellonella Infection Model

**Figure 1:**
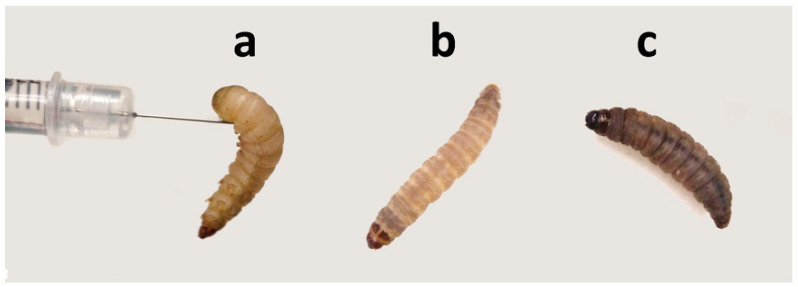
In vivo assays using the *Galleria mellonella* larva model. Inoculation by injection of different bacteria inoculum (**a**), healthy larva (**b**), and dead larva (**c**) as a result of *Klebsiella pneumoniae* infection.

Ten *K. pneumoniae* isolates (Table S1) with reduced sensitivity to carbapenems were obtained from different clinical specimens. To investigate the virulence of *K. pneumoniae* isolates in vivo, we used the *G. mellonella* infection model (Figure 1). In this case, larvae survival rates were measured by injecting inoculums, incubating at 37 °C and recording the survival rate daily for up to three days. In all experiments, control groups with administration of phosphate-buffered saline (PBS) solution resulted in a 100% survival rate.

**Figure 2:**
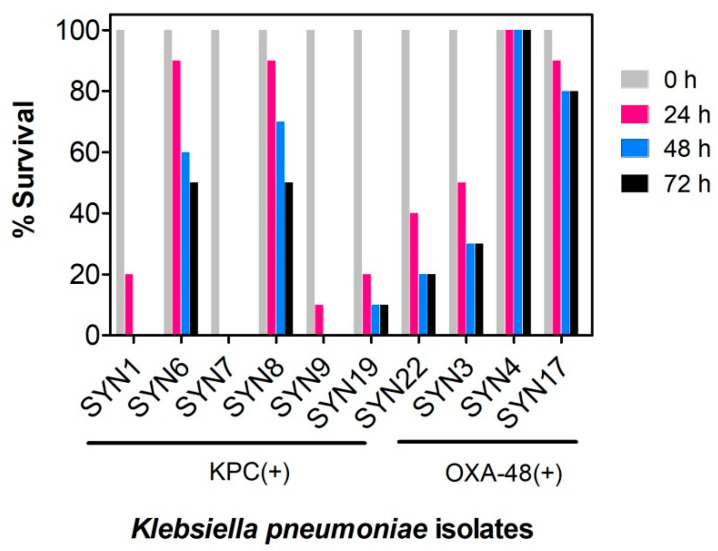
Evaluation of virulence of *Klebsiella pneumoniae* KPC(+) and OXA-48(+) isolates in *Galleria mellonella*. Survival of *G. mellonella* was followed for three days after infection with *K. pneumoniae* KPC(+) and OXA-48(+) with 1 × 10^5^ CFU per larva. Ten larvae were analyzed in each condition and larvae survival was monitored daily. In all cases, no larvae death was observed upon administration of PBS (control). KPC(+) isolates: SYN1, SYN6, SYN7, SYN8, SYN9 SYN19 and SYN22; OXA-48(+) isolates: SYN3, SYN4 and SYN17.

Most larvae were found healthy following infection with 1 × 10^4^ CFU (colony-forming unit) of each isolate per larva (Figure S1), which suggests that at this bacterial density the humoral immunity of the insects is enough to produce an adequate response to the infection. However, the larvae survival was significantly altered upon increase of infection ratio (1 × 10^5^ CFU per larva). As shown in Figure 2, the virulence of isolates varied widely, with some KPC(+) isolates promoting total larvae mortality (e.g., SYN7 KPC clinical isolate). These differences in virulence are in accordance with data reported for patients suffering from *K. pneumoniae* KPC(+) infections [20], confirming the reliability of the *G. mellonella* infection model in reporting pathogenicity differences between all *K. pneumoniae* isolates. This infection model (*G. mellonella* infected with 1 × 10^5^ CFU *K. pneumoniae* per larva) is now well established in our lab, and we believe that will be very helpful in future development of *K. pneumoniae* therapeutics.

### 2.2. Antimicrobial Activity of L-OEI-h

The oligomer L-OEI-h was synthesized following our reported protocol [19]. We evaluated the antimicrobial activity of L-OEI-h against *K. pneumoniae* isolates by determination of the minimum inhibitory concentration (MIC) and the minimum bactericidal concentration (MBC). MIC is defined as the lowest drug concentration that prevents visible growth of bacteria. The MBC is the lowest concentration of an antimicrobial agent required to kill ≥99.9% bacteria over an extended period (18–24 h). The antimicrobial assays were conducted according with CLSI guidelines in Mueller Hinton broth (MHB), a nutrient rich bacterial growth medium. The obtained results for the different isolates are shown in Table 1. We included other Gram-negative bacteria (*Pseudomonas aeruginosa* PAO and *E. coli AB1157*) and a Gram-positive strain (*S. aureus* MRSA JE2) as control strains. Except for SYN7 KPC, which is also the most virulent isolate, L-OEI-h displayed good antibacterial activity against Gram-negative strains with particular relevance for *P. aeruginosa* PAO and *E. coli* AB1157. The MIC and MBC values were almost identical, which is indicative that the oligomer exerts a bactericidal activity.

We previously demonstrated that L-OEI-h is also able to target some Gram-positive bacteria with low efficiency [19], as demonstrated here for methicillin-resistant *S. aureus* JE2 (MIC > 915 μg/mL).

### 2.3. Biocompatibility Studies

**Figure 3:**
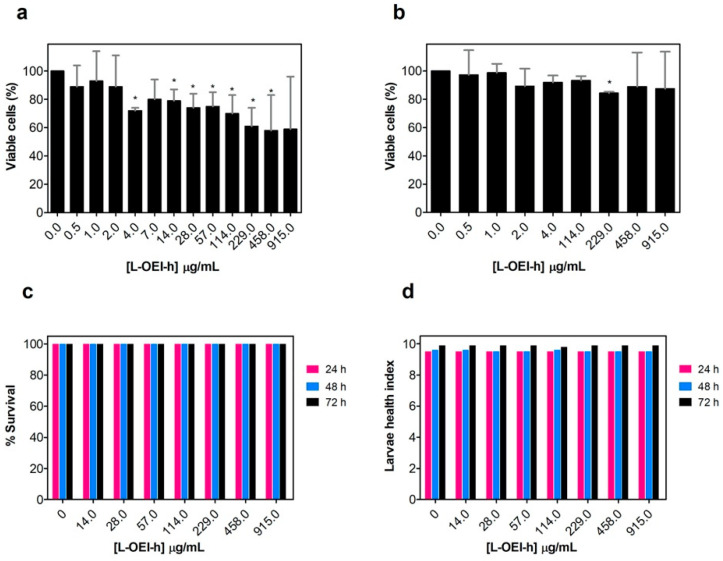
Evaluation of linear oligoethyleneimine hydrochloride (L-OEI-h) toxicity and biocompatibility using in vitro mammalian cells (L929 mouse and A549 human epithelial cells) (**a**,**b**) and in vivo assays (*Galleria mellonella* larvae) (**c**). The health index scores of wax worms injected with L-OEI-h was also evaluated (**d**). Asterisks (*) represent statistical significance in *t*-student tests (*p* < 0.05) compared to the untreated samples.

Some membrane-lytic agents are known to display selectivity towards bacterial membranes, allowing for elevated antibiotic activity and low toxicity to mammalian cells [21]. The differences in composition and lipid arrangement in bacterial and mammalian cell membranes can support the selectivity observed in these antimicrobial agents [21,22]. Here, we evaluated the cytotoxicity of L-OEI-h using in vitro (L929 mouse fibroblasts) and in vivo models (*G. mellonella* larvae) (Figure 3).

The polycationic oligomer compound has very little cytotoxicity against mammalian cell lines, even at the highest dose tested against clinical isolates (915 μg/mL, Figure 3a,b). The larvae were injected with 5 μL of different concentrations of L-OEI-h, and then incubated in Petri dishes at 37 °C and daily scored for survival. Up to a concentration of 915 μg/mL, all larvae were found healthy for a three-day period (Figure 3c). To obtain more differences in larvae health, we also determined the health index scores (Figure 3d), which scores four main parameters: larvae activity, cocoon formation, melanization and survival. The injection of the larvae with L-OEI-h even after 72 h resulted in high health index scores. The higher activity and more cocoon formation are regularly associated to a healthier wax worm [23].

All experiments included a control group injected only with a PBS solution. Overall, our results corroborate the biocompatibility of L-OEI-h.

### 2.4. Exploring the L-OEI-h Mechanism of Action

Cationic polymers are expected to target the microbial cell surface, via binding to negatively charged components, and to disrupt the cytoplasmic membrane [24]. A fast-killing kinetics is associated with surface membranolytic processes, while slow kinetics is usually associated with activation of intracellular processes [25,26,27,28]. In this sense a time-kill assay based on traditional colony count was carried out to discriminate between fast and slow kinetics of antibacterial activity.

**Figure 4:**
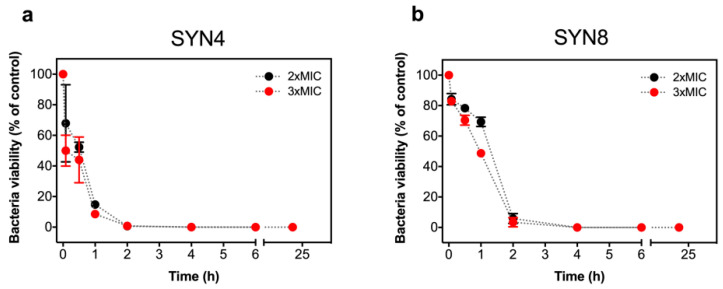
Killing kinetics of *Klebsiella pneumonia* SYN4 OXA-48 (**a**) and SYN8 KPC (**b**) clinical isolates induced by L-OEI-h. The killing kinetics was evaluated with a colony count assay using two different oligomer concentrations, 2 × MIC and 3 × MIC.

Time-kill curves of a selected OXA-48(+) (SYN4 OXA-48) and KPC(+) isolates (SYN8 KPC) were obtained in the presence of two different oligomer concentrations. As shown in Figure 4 and Figure S2, after the addition of L-OEI-h, the number of *K. pneumoniae* viable and culturable colonies decreases significantly in the first 30 min of treatment, with almost complete bacterial removal achieved at 2 h treatment. This observation illustrates a possible fast L-OEI-h bactericidal activity. For other Gram-negative bacteria (e.g., *E. coli* AB1157 [19]), L-OEI-h had a very fast killing effects (within 5 min). In both cases, the verified fast activity is supportive of a surface membranolytic mechanism of action for L-OEI-h.

The higher antimicrobial activity of L-OEI-h against Gram-negative bacteria (e.g., *E. coli* AB1157 vs. *S. aureus* NCTC8325-4 [19]) may indicate that the presence of lipopolysaccharides (LPS) in the outer leaflet of the outer membrane of Gram-negative may facilitate the initial binding of the compound. Antibacterial cationic polymers are expected to permeabilize bacterial membrane by one of two possible mechanisms: (i) Perpendicular insertion in the membrane followed by pore formation and membrane permeabilization/depolarization, or (ii) accumulation at the membrane surface until a certain threshold concentration is achieved, which leads to membrane disruption and cell lysis. 

To further characterize the mechanism of action of L-OEI-h, studies with membrane mimetics were carried out. In these simplified membrane models, size, geometry, and composition can be tailored with great precision. 

**Figure 5:**
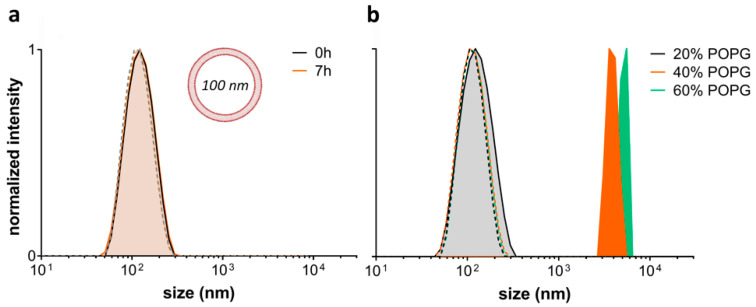
Effect of L-OE-h on model liposomes composed of phosphatidylcholine (POPC) (**a**) and POPC with varying phosphatidylglycerol (POPG) content (**b**). Vesicle sizes were measured by DLS at 25 °C. Dashed lines are the respective controls without polymer.

The effect of L-OEI-h on the membrane was thus, studied using large unilamellar vesicles (LUVs) of controlled lipid composition, serving as mimetics of bacterial and mammalian cell membranes. The bacterial membrane model, with an overall negative charge, was composed of different ratios of phosphatidylcholine (POPC) and phosphatidylglycerol (POPG), while the healthy mammalian plasma membrane model contained only POPC [20]. POPC zwitterionic liposomes are not affected by the addition of L-OEI-h, but higher amount of negatively charged lipids (>20%) led to the formation of large clusters within a few seconds (Figure 5). We hypothesize that the formation of large clusters (>100 nm) is consistent with the binding/accumulation of the polymer in membranes with high negatively charged lipids. 

**Figure 6:**
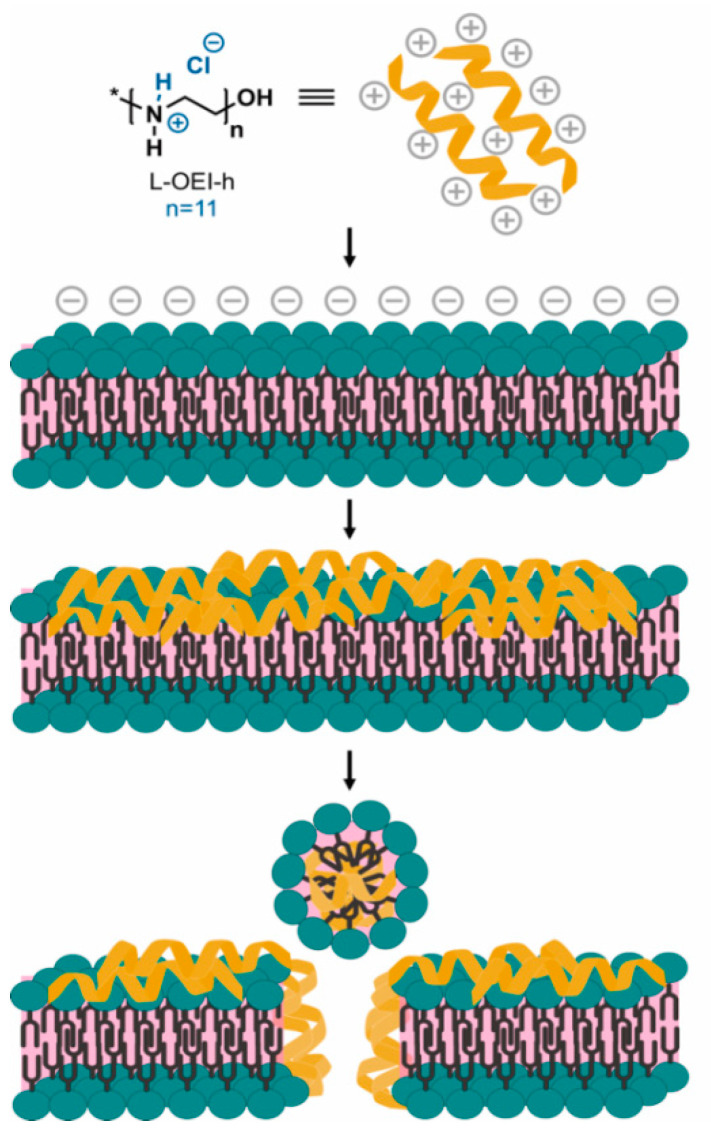
Hypothetical mechanism of action of L-OEI-h (model II) towards bacterial cell membranes. The accumulation of the polycationic oligomer at the membrane surface, due to electrostatic interactions, results in an oligomer threshold concentration capable of cell disruption and lysis.

Upon electrostatic binding of L-OEI-h to anionic lipid vesicles, and after a certain threshold concentration, the oligomer induces vesicle aggregation and/or vesicle fusion, which explains the appearance of a LUV population larger in size (Figure 5b). Vesicle aggregation and fusion are not unusual occurrences, being also observed in the case of cationic peptides addition to anionic vesicles [29]. Ultimately, accumulation of L-OEI-h in the membrane may induce micelle formation or the formation of transient pores, as illustrated for the mechanism of action of some antimicrobial peptides (e.g., [29]). In both cases membrane disintegration is a consequence of these events (Figure 6). 

## 3. Discussion

Bacterial infections are becoming a major human health problem. Resistance towards antibiotics is becoming increasingly common, including to the ones only reserved for the treatment of severe infections. The World Health Organization has recently included *Klebsiella* in the critical list of microorganisms for which new therapeutics are urgently needed [30]. In the light of this demand, in this study we examine the sensitivity of *G. mellonella* to *K. pneumoniae* isolates that are resistant to last resources antibiotics and focused our attention towards a polycationic oligomer [19], a synthetic mimic of host defense peptides (HDPs), as a novel treatment for MDR *K. pneumonia* infections.

HDPs are a class of innate immunity components expressed by all multicellular organisms [31]. It is believed that their function is, in part, to kill invasive cells without prejudice to the host and without presenting itself as a stress agent for the development of resistance traits [31]. The discovery of HDPs was accompanied by the development of disinfectant polymers, which in the late 1990s led to HDP-mimicking polymers [32]. Although antimicrobial peptides (AMPs)/HDPs are an excellent alternative to conventional antibiotics, cheap and scalable bioprocessing is not yet available [33]. Additionally, AMPs are poorly stable in vivo due to protease liability.

In this way, HDP-mimicking polymers are considered a more cost-effective and stable alternative to HDP/AMP [31]. The relationship between structure and activity of HDP-mimetic polymers relies on two main design principles: (i) Hydrophobic/hydrophilic component ratio and (ii) presence of a structured cationic group. It was demonstrated that a poly(methacrylate) random copolymer with 40% methyl side chains (hydrophobic segment) and 60% aminoethyl side chains (hydrophilic segment) show potent antibacterial activity and low hemolytic activity [34].

The effect of primary amine groups, instead of traditional quaternary ammonium salt (QAS), was already investigated [35]. For the same type of polymer, primary amines outperformed in comparison with tertiary or quaternary amines in terms of antimicrobial activity and toxicity. Following this study, it was found that primary ammonium groups can form a stronger complex with phospholipid headgroups when compared to QAS analogues [36]. Additionally, the effect of amine groups density is quite relevant since increasing the amine density by monomer unit enhances the polymers efficacy and decreases hemolysis in great extent [37].

**Figure 7:**
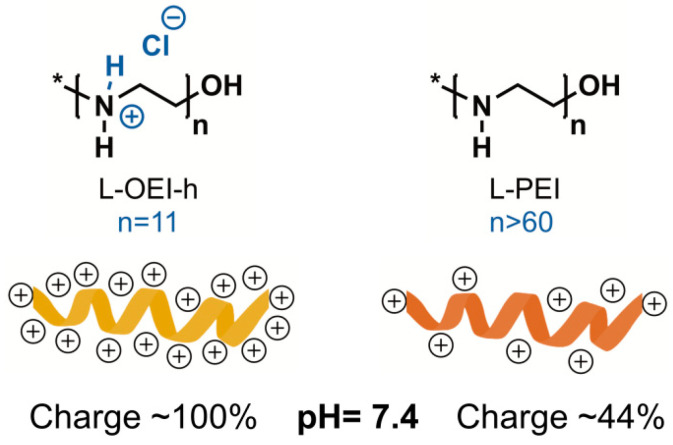
Comparison of positive charge density between linear oligoethyleneimine hydrochloride (L-OEI-h) and linear polyethyleneimine (L-PEI) at physiological pH.

The synthetic polymer polyethyleneimine (PEI), due to its intrinsic features, is regarded as a good alternative to fight antibiotic resistant organisms. The abundance of reactive amine groups in the backbone allows post-modifications to display both hydrophobicity and a positive charge density, primary requirements for a good antimicrobial activity [38]. In previous studies, we found that L-OEI-h, a PEI analogue, is a very effective biocidal oligomer [19], whose activity may be attributed to high positive charge density (hydrochloride salt, quaternary nitrogen atoms), if compared with commercial L-PEI (non-quaternary nitrogen atoms) [39] (see Figure 7). As we and others demonstrated, a higher positive charge density leads to higher antimicrobial efficacy [40]. 

In this work, we evaluated the antimicrobial activity of L-OEI-h against a variety of *K. pneumonia* strains. The antimicrobial efficiency is attributed to favorable electrostatic interactions between the polycationic oligomer (having *ca.* +11 formal net charge, one positive charge per monomer unit) and the anionic bacterial membrane. This interaction can induce a very fast-bactericidal activity, as demonstrated by us [19]. Such a fast mode of action strongly suggests that membranolytic processes are responsible for antimicrobial activity. Through direct permeabilization of the lipidic membrane, instead of action on a specific cellular target, the probability of bacteria to develop resistance to this treatment is extremely low.

For *K. pneumoniae* clinical isolates, the bactericidal activity of the oligomer does not occur earlier than 30 min, in contrast with what we and others verified for other membrane disruption agents, whereby membrane permeabilization and/or lysis happened within 5 min [19,41,42]. It is likely that, for *K. pneumoniae* clinical isolates, L-OEI-h cannot diffuse so efficiently (or diffuses slowly) through the bacterial cell wall to reach the plasma membrane. This could be associated with the fact that the capsule (composed of extracellular polysaccharides) of *K. pneumoniae* is more “robust” than what was verified for other gram-negative bacteria, and, in this sense, constitutes an efficient barrier against several antimicrobial agents [43,44] including HDPs. Despite this, L-OEI-h was able to efficiently kill several *K. pneumoniae* clinical isolates (Table 1, Figure 4 and Figure S2).

Importantly, the L-OEI-h antimicrobial action against some *K. pneumoniae* clinical isolates such as SYN4 OXA-48 and SYN9 KPC (a highly virulent clinical isolate) is verified under a possible therapeutic window, since L-OEI-h induces low cytotoxicity against in vitro mammalian cell lines (<80% cell viability [45] within statistical error) and, more importantly, no visible cytotoxicity was detected against the in vivo model (*G. mellonella*). As shown here, low toxicity in these models is likely associated with reduced interaction with the plasma membrane of eukaryotic cells. Liposomes with a lipid composition mimicking the outer leaflet of eukaryotic plasma membranes were not affected by the presence of L-OEI-h (Figure 5a). On the other hand, liposomes rich in phosphatidylglycerol, mimicking bacterial membrane lipid composition, showed dramatic aggregation upon interaction with this compound. Large unilamellar vesicles (LUVs) were used in these studies as their size is more amenable to DLS resolution. Other membrane models, such as multilamellar vesicles (MLVs), due to their multi-layered character, prevent polymer interactions with the internal bilayers (that could mask possible effects occurring only in the outer membrane), while small unilamellar vesicles (SUV)’s small size results in membranes with excessive curvature that do not mimic bacterial membranes. On the other hand, very large vesicles such as giant unilamellar vesicles (GUVs) have a less controlled lipid concentration/size and thus frequently show significant vesicle heterogeneity within the same sample.

Although we obtained promising data for some *K. pneumoniae* isolates, we verified that in some cases MIC and MBC values were still high (if compared with the results obtained for the Gram-negative control strains). The polymer design and the polymer-membrane interactions are crucial issues for bacterial infection eradication. In a recent study, linear and branched PEI polymers were found to have very similar MIC values, while ε-polycaprolactone showed superior broad-spectrum antimicrobial properties over L-PEI [46]. Hence, in future work we will consider the use of a coarse-grain (CG) molecular dynamics model of L-OEI-h to better understand its interactions with bacterial and mammalian membranes. This study will allow the identification of key oligomer-membrane interactions which could lead to enhanced polymer antimicrobials.

## 4. Materials and Methods

### 4.1. Synthesis of Linear Oligoethyleneimine Hydrochloride (L-OEI-h)

The preparation of L-OEI-h was made in two steps following our previous protocol. First, linear 2-ethyl(2-oligooxazoline) (OEtOx) was synthesized by cationic ring-opening polymerization (CROP) in supercritical carbon dioxide, a green polymerization methodology [47]. The living OEtOx polymer was terminated with water and isolated as a brownish sticky oil. Next, OEtOx was hydrolyzed overnight using a HCl 5M solution. After this period, the precipitated solid was filtered and washed with acetone to obtain L-OEI-h as an off-white solid in quantitative yield. After vacuum drying, L-OEI-h is ready to use [19].

### 4.2. Clinical Isolates Collection and Identification

The KPC and OXA-48-positive carbapenems-resistant *K. pneumoniae* clinical isolates were collected from patients treated in medical care units (Lisbon, Portugal). All strains were identified with matrix-assisted laser desorption/ionization time-of-flight (MALDI-TOF) spectrometry [48,49] using the VITEK MS system (bioMérieux). Briefly, inoculation loops were used to select and smear the isolates onto the sample spots/target slide. Then 1 μL VITEK mass spectrometry α-cyano-4-hydroxycinnamic acid (MS-CHCA) matrix was applied over the sample and air dried (1–2 min). The target slide was loaded into the VITEK MS system to acquire the mass spectra of whole bacterial cell protein (which is mainly composed of ribosomal proteins). Then, the mass spectra acquired for each sample were compared to the mass spectra contained in the database.

In addition, the following antimicrobials were included in the microorganism’s characterization: β-lactams (ceftazidime, cefepime, cefuroxime/axetil, amoxicillin/clavulanate, ticarcillin/clavulanate and piperacillin/tazobactam), carbapenems (meropenem, ertapenem), aminoglycosides (gentamicin, amikacin), nitrofurantoin fluoroquinolones (ciprofloxacin), polymyxin (colistin), fosfomycin, trimethoprim/sulfamethoxazole. The production of carbapenemases in these strains is evidenced through the antibiotic resistance profile and the type of carbapenemase (OXA-48-like, KPC, NDM or VIM) was identified through an immunochromatographic method (RESIST-4 O.K.N.V., Coris). CASFM-EUCAST 2016-defined breakpoints for *Enterobacteriaceae* were used to interpret susceptibility data for *K. pneumoniae* (http://www.sfm-microbiologie.org).

From pure culture on MacConkey agar plates, all identified *K. pneumoniae* isolates were transferred to 1.5 mL Eppendorf tubes contain Luria–Bertani (LB) broth with 20% (*v*/*v*) glycerol and were maintained at −80 °C for long-term storage. For the antimicrobial activity studies, bacterial cultures were inoculated in Mueller-Hinton broth (MHB) (Difco), at 37 °C. Tryptic soy agar (TSA) agar plates (bioMérieux, Marcy l’Etoile, France) were used to subculture *K. pneumoniae* isolates, *P. aeruginosa* PAO and *E. coli* AB1157. *S. aureus* MRSA JE2 was streaked on Columbia agar +5% sheep blood plates (COS, bioMérieux, Marcy l’Etoile, Auvergne-Rhône-Alpes, France) and grown overnight at 37 °C. *P. aeruginosa* PAO, *E. coli* AB1157 and *S. aureus* JE2 were used as reference strains. The antimicrobial activity of L-OEI-h against *E. coli* AB1157 was previously investigated by us [19].

### 4.3. Galleria mellonella Infection Model

*G. mellonella* wax moth larvae were reared in our lab at 25 °C in the dark, from egg to last-instar larvae, on a natural diet (beeswax and pollen grains). Worms of the final-instar larval stage, weighing 250 ± 25 mg, were selected for the experiments. The *G. mellonella* survival experiment was adapted from previous studies with small changes [11,14]. Briefly, all *K. pneumoniae* isolates were grown overnight in TSA plates. Then, the assay was carried out by preparing two distinct inoculums of 2 × 10^6^ and 2 × 10^7^ CFU/mL in PBS. Using a hypodermic microsyringe, the larvae were injected with 5 μL of each bacterial suspension via the hindmost left proleg, previously surface sanitized with 70% (*v*/*v*) alcohol. Different groups were used (*n* = 10 each)—larvae injected with PBS to monitor the killing due to injection trauma (control) and larvae injected with *K. pneumoniae* isolates. After inoculation, larvae were kept in Petri dishes and maintained in the dark at 37 °C for 72 h. The larval survival was assessed daily during that period, and caterpillars were considered dead based on the lack of mobility in response to touch. Each larva was also scored daily to the *G. mellonella* health index, which scores four main parameters: Larvae activity, cocoon formation, melanization and survival, as described in [23].

All experiments were performed using a minimum of two independent experiments.

### 4.4. Antimicrobial Activity

#### 4.4.1. Minimum Inhibitory Concentration (MIC) Determination

To determine the MIC values, the bacterial suspension was initially adjusted to a concentration of 1 × 10^6^ CFU/mL in MHB (according to CLSI guidelines) [50]. On a 96-well plate (Orange Scientific, Braine-l’Alleud, Belgium), two-fold serial dilution (in MHB) of L-OEI-h was then added to each well that contained the bacterial inoculums (dilution 1:1). Final bacteria inoculum in each well were diluted to 5 × 10^5^ CFU/mL. The 96-well tissue culture plates were incubated for 18–20 h at 37 °C. All experiments were performed using a minimum of three independent experiments performed with three technical replicates each.

#### 4.4.2. Minimum Bactericidal Concentration (MBC) Determination

The MBC values were determined by the traditional colony count assay [51]. At the end of the MIC assay, 20 μL samples from each well (corresponding to ½ MIC, MIC, 2 × MIC, 3 × MIC of L-OEI-h) were transferred to a new 96-well plate and successively diluted (10-fold) in MHB. Then, each dilution was sub-cultured in TSA plates. After incubation at 37 °C for 24 h, the resultant viable colonies were counted. All experiments were performed using a minimum of three independent experiments performed with three technical replicates each.

#### 4.4.3. In Vitro Time-Kill Curves

Time-kill curves of L-OEI-h were determined according to literature [52,53]. Briefly, a final inoculum of 1 × 10^6^ to 1 × 10^7^ CFU/mL was exposed to distinct doses of L-OEI-h and incubated at 37 °C. Aliquots at specified time points were taken; 10-fold dilutions of each well were prepared and plated onto a TSA plate for CFU enumeration. TSA plates were incubated for 24 h at 37 °C and bacterial colonies were counted. Viable cells (CFU/mL) are reported here as percentage of the control (bacterial suspension without L-OEI-h exposition). From the number of bacterial colonies obtained, viable bacteria (in CFU/mL) are reported as percentage of the control. All experiments were performed using two independent experiments with three technical replicates each.

### 4.5. Biocompatibility Assays

#### 4.5.1. MTT Viability Assay

L-929 and A549 cell lines were cultured in T-75 cell culture flasks (Filter caps) using Dulbecco’s modified Eagle’s medium (DMEM, Catalog number 41966-029) supplemented with 10% fetal bovine serum (Thermo Fisher Scientific, Catalog number 10500-064, heat inactivated) and 1% penicillin-streptomycin (Thermo Fisher Scientific, catalogue number 15140-122) and maintained in a humidified atmosphere with 5% CO_2_ at 37 °C. All cell culture lines were maintained with routinely subcultures (using TrypLE Express without phenol red, GIBCO™, for chemical detaching). Mammalian cell lines were counted with a hemocytometer.

The MTT assay was used to detect changes on metabolic activity of mammalian cells [54]. Briefly, the L-929 and A549 cell lines were seeded in 96-well flat-bottomed polystyrene plates with a density of 1 × 10^4^ cells/well and left to adhere overnight in a CO_2_ incubator (5%) at 37 °C. After 24 h, the cell medium was discarded and replaced with fresh medium containing different concentrations of L-OEI-h. Cells were then incubated for a period of 24 h at 37 °C in a humidified 5% CO_2_ incubator. After this incubation period, the medium was discarded and 20 µL of MTT (5 mg/mL) were added to each well together with 100 µL of fresh DMEM and incubated at 37 °C for 3.5 h. The formazan crystals formed in the wells were dissolved using 150 μL of MTT solvent (4 mM of HCl, 0.1% of Nondet P-40 in isopropanol). The formation of formazan was monitored by measuring the absorbance at 590 nm in a microplate reader (BMG Labtech, Polar Star Optima). Cell viability was determined relatively to the untreated sample after correcting the data with the negative control.

All experiments were performed using a minimum of two independent experiments with three technical replicates each.

#### 4.5.2. *Galleria mellonella* Toxicity Assay

The L-OEI-h toxicity was also evaluated in the larvae infection in vivo model. The *G. mellonella* killing assays were based on the above descriptions with small modifications. L-OEI-h doses were prepared and injected into the larvae hindmost left proleg. The larvae survival was assessed daily during a period of 72 h. A control group was also included in the assay. Two independent experiments were performed.

### 4.6. Exploring the L-OEI-h Mechanism of Action

#### Liposome Preparation

Large unilamellar vesicles (LUVs), with 100 nm of diameter, were prepared by extrusion of multilamellar vesicles [54]. The liposomes were prepared according to methods previously described [55]. Briefly, lipid mixtures composed of adequate amounts of lipids (POPC and POPG) were prepared in chloroform to a final lipid concentration of 2 mM. The solvent was slowly vaporized under a nitrogen flux and the resulting lipid film was left in vacuum for 3 h to ensure the complete removal of chloroform. Afterwards, the lipid was resuspended in 2 mL of DPBS (Thermo Fisher Scientific) and freeze–thaw cycles (liquid nitrogen/water bath at 60 °C) were performed to re-equilibrate and homogenize the samples. LUVs were finally obtained by extrusion of the solutions at 50 °C with an Avanti Mini-Extruder (Merck, Darmstadt, Germany) using 100 nm pore size polycarbonate membranes. All lipid stock solutions were prepared in chloroform and the respective concentrations were determined by the colorimetric quantification of inorganic phosphate.

Liposome size was determined by dynamic light scattering (DLS) using a Nanosizer ZS (Malvern Instruments). The POPC/POPG vesicles were incubated with L-OEI-h for 5 min. Data was collected at 25 °C and a backscattering angle of 173°. Two independent experiments were performed.

## Figures and Tables

**Table 1:** Antimicrobial activity of linear oligoethyleneimine hydrochloride (L-OEI-h) against *Klebsiella pneumoniae* isolates, control bacterial strains (*P. aeruginosa* PAO, *E. coli* AB1157) and the methicillin-resistant *Staphylococcus* strain *S. aureus* JE2.

Clinical Isolate	MIC (μg/mL)	MBC (μg/mL)
SYN1 KPC	458	458
SYN3 OXA-48	915	>915
SYN4 OXA-48	458	915
SYN6 KPC	915	915
SYN7 KPC	>915	>915
SYN8 KPC	458	458
SYN9 KPC	229	229–458
SYN17 OXA-48	915	915
SYN19 KPC	915	915
SYN22 KPC	915	>915
*P. aeruginosa* PAO	114	114
*E. coli* AB1157 *	90	90
*S. aureus* JE2	>915	>915

* Data acquired in a previous study [13].

## Data Availability

The data presented in this study are available in the article and in the supplementary material.

## Supplementary Materials

The following are available online at https://www.mdpi.com/2079-6382/10/1/56/s1, Figure S1: Evaluation of distinct virulence response of *K. pneumoniae* KPC(+) and OXA-48 (+) isolates in *G. mellonella*. Figure S2: Killing kinetics of *K. pneumonia* SYN4 OXA-48 (a) and SYN8 KPC (b) clinical isolates induced by L-OEI-h. Table S1: *K. pneumonia* clinical isolates origin and the minimum inhibitory concentration (MIC) values of trimethoprim (TM)/sulfamethoxazole (SM) antibiotics.
