# Supplementary material for: Carbapenem-Resistant Klebsiella pneumoniae Clinical Isolates: In Vivo Virulence Assessment in Galleria mellonella and Potential Therapeutics by Polycationic Oligoethyleneimine
Source: Antibiotics (Basel). 2021 Jan 8;10(1):56. doi: 10.3390/antibiotics10010056 (PMC7826767; doi:10.3390/antibiotics10010056)
Supplement: Supplementary file 1 [file antibiotics-10-00056-s001.pdf]

Carbapenem-Resistant *Klebsiella pneumoniae* Clinical Isolates: In Vivo Virulence Assessment in *Galleria mellonella* and Potential Therapeutics by Polycationic Oligoethyleneimine

Dalila Mil-Homens [1], Maria Martins [1], José Barbosa [1], Gabriel Serafim [1], Maria J. Sarmento [2], Rita F. Pires [1], Vitória Rodrigues [3], Vasco D.B. Bonifácio [1,*] and Sandra N. Pinto [1,*]

[1] iBB-Institute for Bioengineering and Biosciences, Department of Bioengineering, Instituto Superior Técnico, Universidade de Lisboa, Av. Rovisco Pais, 1049-001 Lisboa, Portugal; dalilamil-homens@tecnico.ulisboa.pt (D.M-H.); mald.martins@campus.fct.unl.pt (M.M.); josembarbosa@tecnico.ulisboa.pt (J.J.); ritafpires@tecnico.ulisboa.pt (R.F.P.)
[2] J. Heyrovský Institute of Physical Chemistry of the Czech Academy of Sciences, Dolejškova 3, 18223 Prague 8, Czech Republic; maria.sarmento@jh-inst.cas.cz
[3] Secção de Microbiologia, Laboratório SYNLAB - Lisboa, Grupo SYNLAB Portugal, Av. Columbano Bordalo Pinheiro, 75 A, 2º andar, 1070-061 Lisboa, Portugal; vitoria.rodrigues@synlab.pt

* Correspondence: sandrapinto@ist.utl.pt (S.N.P); vasco.bonifacio@tecnico.ulisboa.pt (V.D.B.B.)

Table S1. *Klebsiella pneumonia* clinical isolates origin and the minimum inhibitory concentration (MIC) values of trimethoprim (TM)/sulfamethoxazole (SM) antibiotics.

Code	Resistance*	Origin	MIC TM/SM (μg/mL)
SYN1	KPC	Biopsy	≥320
SYN3	OXA-48	Rectal excrement	≥320
SYN4	OXA-48	Urine	≥320
SYN6	KPC	Urine	≥320
SYN7	KPC	Purulent excrement	≥320
SYN8	KPC	Pus	≥320
SYN9	KPC	Urine	≤20
SYN17	OXA-48	Hemoculture	≥320
SYN19	KPC	Perianal	≥320
SYN22	KPC	Pus	≥320

*All OXA-48 isolates are resistant to several antibiotics including TM/SM and ertapenem. KPC isolates are also resistant to several antibiotics including ertapenem, meropenem and TM/SM. In the case of TM/SM, SYN9 KPC was the only exception.

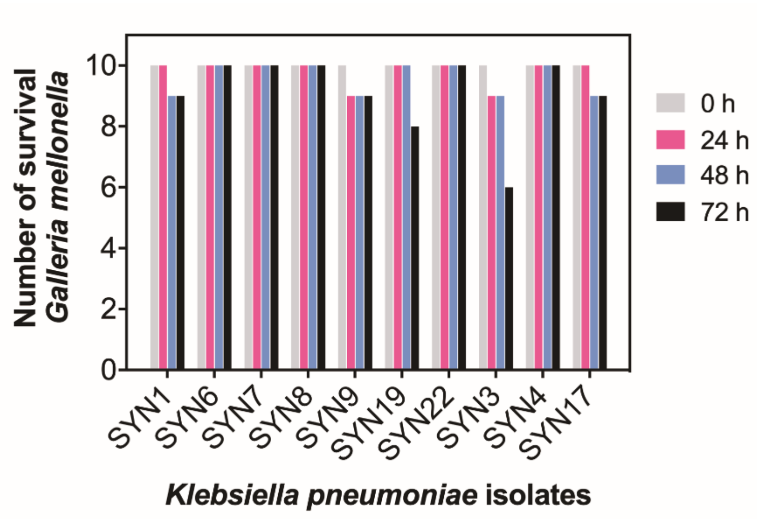

Figure S1. Evaluation of distinct virulence response of *Klebsiella pneumoniae* KPC(+) and OXA-48 (+) isolates in *G. mellonella*. The survival of *G. mellonella* was followed for 3 days after infection with *K. pneumoniae* KPC(+) and OXA-48(+) using 10^4 CFU *per* larva. Ten larvae were analysed in each condition and larvae survival was daily monitored. In all cases, no larvae death was observed upon administration of PBS (control).

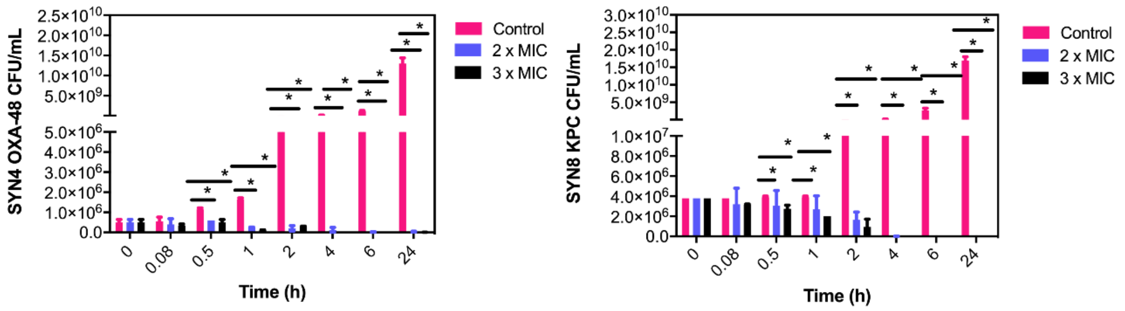

Figure S2. Killing kinetics of *Klebsiella pneumonia* SYN4 OXA-48 (a) and SYN8 KPC (b) clinical isolates induced by linear oligoethyleneimine hydrochloride (L-OEI-h). The killing kinetics was evaluated with a colony count assay using two different polymer concentrations, 2 × MIC and 3 × MIC. Asterisks represent statistical significance in *t*-student test ($p<0.05$). Error bars represent standard deviation resultant from two biological replicates. Results are reported in CFU/mL.
